# *Plasmodium falciparum* Plasmepsin 2 Duplications, West Africa

**DOI:** 10.3201/eid2408.180370

**Published:** 2018-08

**Authors:** Juliana Inoue, Miguel Silva, Bakary Fofana, Kassim Sanogo, Andreas Mårtensson, Issaka Sagara, Anders Björkman, Maria Isabel Veiga, Pedro Eduardo Ferreira, Abdoulaye Djimde, José Pedro Gil

**Affiliations:** Uppsala University, Uppsala, Sweden (J. Inoue, A. Mårtensson, J.P. Gil);; University of Minho, Braga, Portugal (M. Silva, M.I. Veiga, P.E. Ferreira);; University of Science, Techniques, and Technologies of Bamako, Bamako, Mali (B. Fofana, K. Sanogo, I. Sagara, A. Djimde);; Karolinska Institutet, Stockholm, Sweden (A. Björkman, J.P. Gil);; Universidade de Lisboa, Lisbon, Portugal (J.P. Gil)

**Keywords:** malaria, piperaquine, resistance, Plasmodium falciparum, plasmepsin 2, Pfpm2, dihydroartemisinin-piperaquine, DHA-PPQ, West Africa, vector-borne infections, moquitoborne diseases

## Abstract

Dihydroartemisinin/piperaquine (DHA/PPQ) is increasingly deployed as an antimalaria drug in Africa. We report the detection in Mali of *Plasmodium falciparum* infections carrying plasmepsin 2 duplications (associated with piperaquine resistance) in 7/65 recurrent infections within 2 months after DHA/PPQ treatment. These findings raise concerns about the long-term efficacy of DHA/PPQ treatment in Africa.

Artemisinin combination therapy has been the cornerstone of malaria control in sub-Saharan Africa for the past 10 years and is typically represented by artemether/lumefantrine and artesunate/amodiaquine. Because of the notorious capacities of *Plasmodium falciparum* to develop drug resistance, many antimalarial programs have recently included dihydroartemisinin/piperaquine (DHA/PPQ) as a second-line antimalarial drug. This decision is sensible, considering the recent reports of substantially decreased artemether/lumefantrine cure rates in some regions, signaling a potential focus of lumefantrine resistance (*1*).

DHA/PPQ has shown near-perfect efficacy levels in clinical trials conducted in Africa; the combination also has been proposed as a tool for intermittent preventive approaches (*2*). Unfortunately, full *P. falciparum* resistance to DHA/PPQ treatment has been reported recently in Cambodia (*3*,*4*). These events were directly associated with increased copy number variations (CNVs) in the plasmepsin system, including the *pfpm2* gene (PF3D7_1408000) coding for the food vacuole enzyme plasmepsin II, which is speculated to be a major piperaquine target.

CNV is generally considered as emerging at relatively rapid mutation rates (a rate several orders of magnitude higher compared with that of single-nucleotide polymorphisms [*5*]) and is able to generate substantial diversity (*6*). Therefore, preexisting *pfpm2* duplications in Cambodia might have been rapidly selected by DHA/PPQ, aided by a less effective protective action of the artemisinin derivative (*7*). Such a scenario suggests that this mutation may already be present in Africa.

To investigate this possibility, we analyzed a subset of archived *P. falciparum* DNA samples from clinical infections, derived from a set of large, multicenter comparative artemisinin combination therapy efficacy trials conducted in West Africa by the West African Network for Antimalarial Drugs (*8*). These trials, performed during October 2011–February 2016 in Mali, Burkina Faso, and Guinea, had a randomized double-blind design with a 2-year follow-up for monitoring repeated treatment. Here we focus on the DHA/PPQ trial conducted at the village of Bougoula-Hameau in Mali, located ≈350 km south of the capital city of Bamako, near the border with Burkina Faso. The weekly control follow-up for each episode at Bougoula-Hameau was 63 days, and the DHA/PPQ arm involved a total of 224 patients who were >6 months of age.

We conducted a pilot study analyzing the 96 recurrent infections associated with the shortest interepisode periods, assuming that this subgroup, among whom initiation of recurrent infection ranged from 23 to 65 days posttreatment (Figure), would be the most likely to include *pfpm2* duplications. The study was reviewed and approved by the Ethics Committee of the Faculty of Medicine, Pharmacy, and Odonto-Stomatology, University of Sciences, Techniques and Technology of Bamako.

**Figure:**
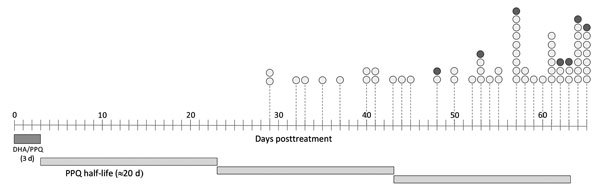
Timeline distribution of *Plasmodium falciparum* *pfpm2* copy number status during post–DHA/PPQ treatment follow-up for artemisinin combination therapy efficacy trials conducted by the West African Network for Antimalarial Drugs, Mali, Burkina Faso, and Guinea, October 2011–February 2016. Dark gray bar highlights the period (3 d) of treatment; lighter, longer gray bars represent PPQ average half-life (≈20 d). Circles represent recurrent infections; white circles indicate 1 *pfpm2* copy, and gray circles indicate 2 *pfpm2* copies. DHA/PPQ, dihydroartemisinin/piperaquine; PPQ, piperaquine.

We determined copy number by using a SYBR-green–based quantitative PCR (ThermoFisher Scientific, Waltham, MA, USA) in a protocol modified from the one previously described by Witkowski et al (*4*). We used the *P. falciparum* β-tubulin gene as the internal nonduplicated standard and the 3D7 clone as a parallel 1 copy control. We ran the quantitative PCR thermal cycle at 98°C for 3 min, followed by 45 cycles at 98°C for 15 s, 63°C for 20 s, and 72°C for 20 s on a C1000 Thermal Cycler (Bio-Rad, Marnes-la-Coquette, France) with the CFX96 Real-Time System (Bio-Rad) detection system. We executed all procedures in triplicate. 

The analysis was conclusive in 65 of the 96 samples. We confirm the presence of 7 infections carrying 2 copies of *pfpm2*, representing ≈10% of the successfully analyzed infections. We did not identify any trend of earlier recurrence associated with this group of infections (Figure), a preliminarily observation that needs to be further explored in a larger sample set.

Our results clearly show that piperaquine resistance–associated *pfpm2* duplications are probably already frequent in Africa, which is of concern given the long half-life of piperaquine (>20 days). In high-transmission areas, this long period of decreasing drug exposure is likely to progressively select less sensitive, potentially *pfpm2* CNV–carrying parasites. Parallel studies conducted in these areas have not detected substantial altered parasite clearance dynamics or K13 mutations associated with artemisinin-derivative therapy (*9*,*10*), indicating that these *pfpm2* duplications are emerging despite the efficacy of dihydroartemisinin. Further studies are urgently needed to clarify the clinical implications of piperaquine resistance and to monitor occurrence in other areas of high malaria transmission in Africa.
